# Isokinetic training of lower extremity during the early stage promote functional restoration in elder patients with disability after Total knee replacement (TKR) - a randomized control trial

**DOI:** 10.1186/s12877-024-04778-9

**Published:** 2024-02-19

**Authors:** Yuan-Yang Cheng, Cheng-Hsu Chen, Shun-Ping Wang

**Affiliations:** 1 Department of Physical Medicine and Rehabilitation, Taichung Veterans General Hospital, Taichung, Taiwan; 2 School of Medicine, National Yang Ming Chiao Tung University, Taipei, Taiwan; 3 Department of Post-Baccalaureate Medicine, College of Medicine, National Chung Hsing University, Taichung, Taiwan; 4 Division of Nephrology, Department of Internal Medicine, Taichung Veterans General Hospital, Taichung, Taiwan; 5 Ph.D. Program in Tissue Engineering and Regenerative Medicine, College of Medicine, National Chung Hsing University, Taichung, Taiwan; 6 Department of Orthopedics, Taichung Veterans General Hospital, Taichung, Taiwan

**Keywords:** Total knee replacement, Isokinetic, Isotonic, Isometric, Timed up and go

## Abstract

**Background:**

Transient progressive weakness and disability of lower limb during the early stage after TKR will increase the risk of fall, but the superior postoperative strength training mode have not been elucidated for functional restoration. This study aimed to compare whether the isokinetic lower limb training is superior to either isotonic or home isometric exercise during early stage after TKR in older people.

**Methods:**

A total of 43 recruited old participants (mean age, 68.40 years old) receiving TKR were divided randomly based on the different four-week training modes into three groups including isokinetic, isotonic, and home isometric exercise (control group). The primary outcome was set as functional performance in terms of Timed Up and Go (TUG) test and the secondary outcomes include the peak torque of knee at 60 and 120 degree/ second, Short-Form 36 Health Survey (SF-36), and Western Ontario and McMaster Universities Arthritis index (WOMAC).

**Results:**

All of the peak torque measurements of the knee improved significantly in both the isokinetic and the isotonic group, but not in the control group. Although isotonic training resulted in more strength gains, a significant enhancement in TUG test was observed in the isokinetic group only (*p* = 0.003). However, there were no significantly improvement of TUG test after training in other two groups. SF-36 and WOMAC improved after training in all three groups, with no significant difference in the degree of improvement between groups.

**Conclusion:**

Isokinetic training for 4 weeks following TKR effectively improved all the outcome parameters in this study, including the TUG test, lower limb strength, and functional scores. However, both isokinetic and isotonic training modes could be recommended after TKR because of no significant difference in the degree of improvement between these two groups.

**Trial registration:**

Clinical trial registration number: NCT02938416.

**Level of evidence:**

I

## Introduction

Osteoarthritis (OA) of the knee is characterized by gradual wear and tear of the articular cartilage lining inside the knee joints. Advances in the stage of knee OA will result in painful lower limb disability and compromise the quality of life of the elderly. With an ageing population worldwide and an increasing prevalence of obesity in many countries, years of life lived with disability due to hip and knee OA increased from 10.5 million in 1990 to 17.1 million in 2010 [1]. Several conservative treatment options for knee OA are available, ranging from lifestyle modification, physical therapy, orthotic devices, analgesic medications, and viscosupplement injections [2]. If conservative treatments are ineffective, total knee replacement (TKR) is the preferred surgical option to relieve pain and improve physical function for older participants with advanced-stage OA knees [3, 4].

Hicks et al. indicated that knee pain accompanied by decreased knee extension strength and standing balance was significantly correlated with the risk of multiple falls in older people [5]. Patients with OA knee frequently presented progressive knee pain and quadriceps weakness [6], and these problems can even be exacerbated during the early stage following TKR [7]. These factors will increase the risk of falling during early stage. Therefore, the importance of rebuilding quadriceps strength cannot be overemphasized for patients undergoing TKR. Early strengthening exercise will be a crucial component of postoperative rehabilitation programs.

The postoperative strengthening exercise of TKR can be divided into three types: isometric, isotonic, and isokinetic training. Among them, isokinetic training is considered to be the most effective way to improve the functional performance of daily activities, although all three training modes improve the lean muscle mass and strength of the lower limbs [8]. During isokinetic training, the real-time torque produced by muscle contraction can be monitored instantaneously and thus serves as a good method to provide visual biofeedback for trainees. Furthermore, isokinetic strengthening exercise is considered a safer method of strengthening muscles, since the trainee can always perform muscle exertion within their pain limit under the same angular velocity, in contrast to isotonic training, which carries a risk of over-training [9].

In the past, isokinetic training has been reported to be an efficient method of rebuilding quadriceps muscle strength for patients with OA knees [10], following anterior cruciate ligament reconstruction [11], and for patients undergoing TKR [12]. However, to date, few studies directly compared the effects of isokinetic, isotonic or home isometric strengthening exercise on recovery of muscle strength, functional performance and life quality among patients after TKR. Therefore, we designed this randomised controlled trial in order to fill this knowledge gap. This current research was aimed to elucidate which kinds of postoperative exercise strengthening are superior to the elder TKR patients during the early stage and we hypothesize that isokinetic training could result in better recovery of functional performance than isotonic or home isometric exercise.

## Methods

### Participants

This was a prospective, single-blinded, randomized controlled study conducted in a tertiary medical center. It was approved by the Institutional Review Board of Taichung Veterans General Hospital (No. CF12251B-8). This clinical trial had been registered on 19/10/2016 (NCT02938416). The inclusion criteria of the enrolled cases in this study are 1. Radiological diagnosis of advanced degenerative OA knee (Kellgren and Lawrence grade 3 or higher). 2. Undergoing a primary TKR without complications in one knee. 3. No significant impairment of the other knee influencing the sequential tests. 4. At least 1 year after the prior TKR on the other knee. 5. Patients who are at the time of 4 weeks after TKR. The exclusion criteria of this study included: 1. Body Mass Index (BMI) > 35 kg/m^2^. 2. Diagnosis of cardiopulmonary or orthopedic diseases that would prohibit the patient from receiving strength training. 3. Evidence of cognitive impairment that would prevent the patient from understanding and completing the questionnaire or training programs used in this study. 4. Diagnosis of neurological diseases or other pain issues in the lower limbs that affect gait pattern. The participants were recruited from the orthopedic outpatient clinics during an appointment 4 weeks after TKR. In order to determine the number of participants needed in this study, we used G*power 3.1.9.7 (Heinrich-Heine-Universität Düsseldorf, Germany) [13] to perform the sample size calculation based on the differences of their functional capacity in a similar isokinetic study on patients with OA knees [14]. With an alpha error of 0.05 and power setting at 0.8, the effect size was calculated to be 0.624, and it was determined that at least 30 participants were needed to achieve sufficient statistical power in this study. From June 2018 to June 2020, a total of 90 patients with an OA knee underwent TKR performed by a single surgeon in our institution. Among them, 39 were excluded by the exclusion criteria, and the remaining 51 patients were randomized into three groups: isokinetic, isotonic, and control groups. In total, 14 patients completed the isokinetic training, 15 completed the isotonic training, and 14 received only home isometric exercise training. During the training period, 8 participants lost follow-up and declined to continue the following strength training. All of them considered the training programs too cumbersome due to frequent transportation assistance from their family members to comply with our training protocol (3 times/week).

**Fig. 1:**
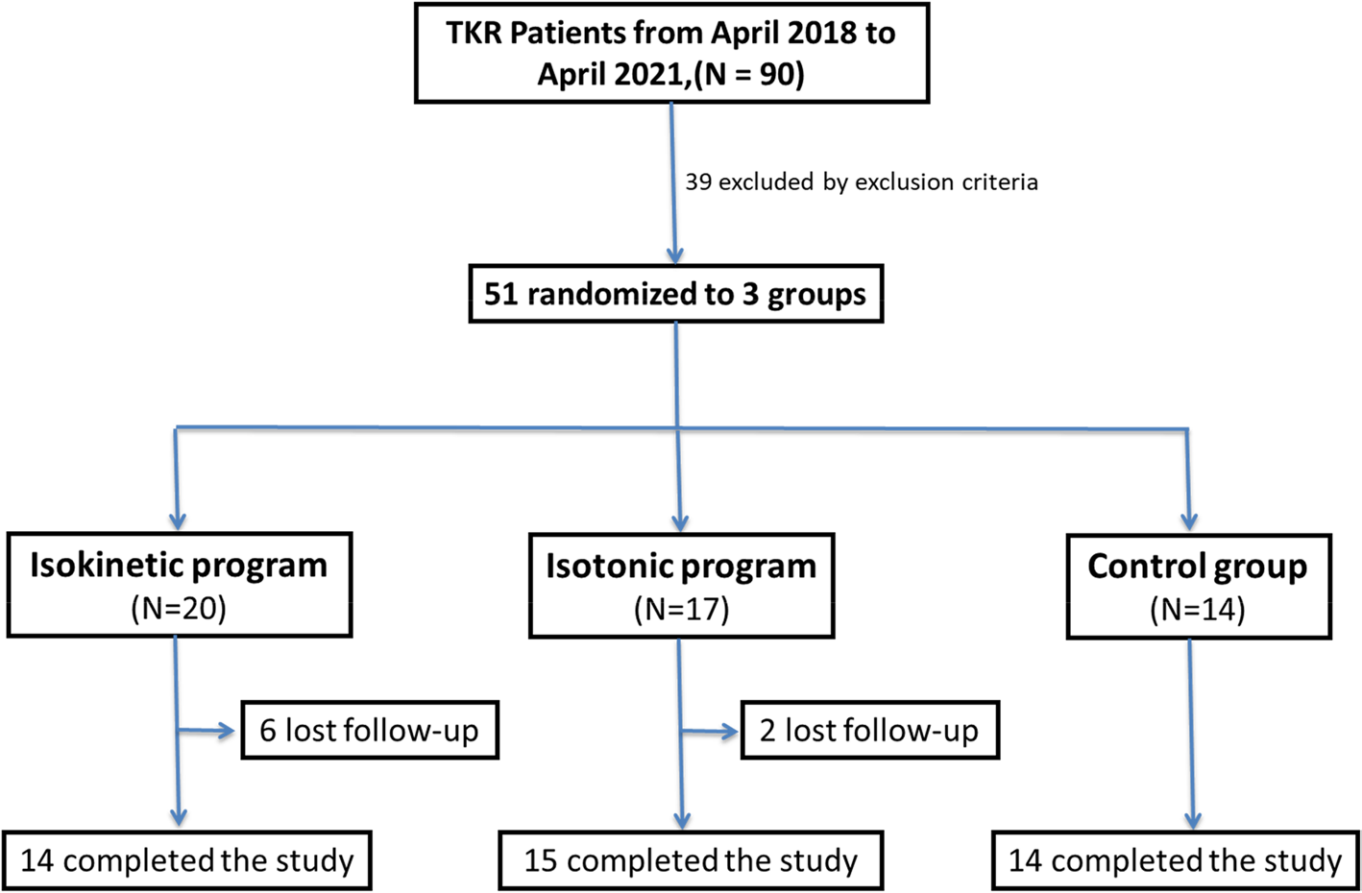
Flowchart of the study

At the end of this research, a total of 43 cases completed the strengthening course. The flowchart of our study is shown in Fig. 1.

### Exercise interventional groups

After providing informed consent, the participants were randomly assigned using the block randomization method into one of three groups by drawing lots: isokinetic (IK) group, isotonic (IT) group, and control group. All participants received the same in-patient rehabilitation programs and outpatient medications during the follow-up period, while patients in the isokinetic and isotonic groups received additional isokinetic and isotonic strengthening training, respectively, at the hospital after enrolling in this study. Patients in the control group, on the other hand, only received further instructions on the execution of home quadriceps isometric strengthening by the physical therapist at outpatient clinic. This exercise involves maintaining the postoperative knee in a straight posture while sitting for 20 seconds and should be performed at least 50 times a day at home.

An isokinetic dynamometer (Biodex Multi-Joint System 3, 187 Biodex Medical Incorporation, New York, USA) was used to perform the isokinetic and isotonic training in this study. In the isokinetic mode of training, participants performed concentric knee flexion and extension in 60 degrees per second (deg./sec) for five repetitions, and then eccentric knee flexion and extension in 60 deg./sec for another five repetitions. A total of three sets of training were done in 1 day, and a three-minute rest was allowed between the training sets. During the isokinetic training, a real-time torque production figure was shown on a screen, providing visual biofeedback so that the participants could see how hard they were performing the exertion. The participants were further encouraged by their physical therapist to push the lever arm as hard as possible during isokinetic training. Furthermore, the isotonic mode of training involved applying a constant resistance at 50% of the peak isometric torque measured at 90 degree of knee flexion. The participants performed knee flexion and extension against the uniform resistance throughout the range of motion for ten repetitions. As in the isokinetic group, a total of three sets of training were done in 1 day with a three-minute rest between the training sets. Both the isokinetic and the isotonic group received strength training 3 days per week, for a total of 4 weeks.

### Outcome measures

The primary outcome of this study was the time to complete the Timed Up and Go (TUG) test. The secondary outcome measures included the peak torque of knee flexion and extension at 60 deg./sec and 120 deg./sec, the Short-Form 36 Health Survey (SF-36), and the Western Ontario and McMaster Universities Arthritis index (WOMAC). These outcome measures were assessed before and after the four-week program of strength training by a research assistant, not the physical therapist who conducted the training programs.

The TUG test was performed with the participant sitting on a chair with the height adjusted so that his thigh was parallel to the floor initially. Afterward, the participant was asked to stand up, walk forward three meters, turn around, walk back to the original chair, and sit down [15]. The total time spent was recorded as the primary objective outcome measure of our study.

Another objective outcome, the peak torque of knee flexion and extension at 60 and 120 deg./sec, was measured using the same isokinetic dynamometer during training. Participants were allowed to familiarize themselves with the testing process. After that, they took a 5-minute rest before the formal testing: five times of maximal knee flexion and extension at the angular speed of 60 and 120 deg./sec with a rest interval of 3 minutes between each exertion. The maximal value among the five exertions was recorded as the outcome measure for knee flexion and extension.

Two subjective questionnaires, the SF-36 and the WOMAC, were also used as outcome measures. SF-36 is a useful clinical tool to evaluate health-related quality of life [16], and consists of 36 questions that are divided into eight subdomains: physical functioning, physical role functioning, bodily pain, general health perception, vitality, social functioning, emotional role functioning, and mental health. The average score of the first four subdomains was used as the score of the physical domain, and the average score of the last four subdomains was used as the score of the mental domain. The two scores above were averaged to represent the overall SF-36 score. The WOMAC is a clinical tool more specific to knee problems [17]. It consists of 24 questions. Among them, five questions are related to pain, two questions are related to stiffness, and the rest are related to physical function. The maximal total score of the WOMAC is 96, which signifies the most severe knee symptoms experienced by the patient.

### Statistical analysis

Predictive Analytics SoftWare (PASW version 18.0, Chicago, IL, USA) was used for statistical analysis in our study. The Shapiro–Wilk test was done for all of the parameters in our study to determine the normality of distribution. If a parameter was normally distributed, analysis of variance (ANOVA) and paired t test were performed. If a parameter did not show a normal distribution, non-parametric tests including the Kruskal–Wallis test and the Wilcoxon Signed Rank test were used instead. We compared the baseline data by ANOVA or Kruskal–Wallis test with Dunn-Bonferroni post hoc tests, and then the pre-test and post-test data of the three groups were compared by paired t test or Wilcoxon Signed Rank test. Finally, the degree of improvement among the three groups was compared by ANOVA or Kruskal–Wallis test. A *p* < 0.05 was considered statistically significant in our study.

## Results

A total of 43 participants completed our study protocol, and the baseline characteristics of our participants are displayed in Table 1. The average age of this cohort was 68.4 ± 8.66 years old. Twenty-nine patients were female, and 14 were male. The most of baseline characteristics were not significantly different among the three groups, except the total score of the WOMAC.

**Table 1:** Baseline characteristics of the participants

	Total (*n* = 43)	Training mode			*p* value	Dunn-Bonferroni post hoc		
		Isokinetic (IK) (*n* = 14)	Isotonic (IT) (*n* = 15)	Control (*n* = 14)		IK vs IT	IK vs. control	IT vs. control
**Age**	68.40 ± 8.66	66.79 ± 10.78	68.47 ± 8.10	69.93 ± 7.09	0.742			
**Sex**					0.204			
Female	29 (67.44%)	12 (85.71%)	9 (60.00%)	8 (57.14%)				
Male	14 (32.56%)	2 (14.29%)	6 (40.00%)	6 (42.86%)				
**Affected joint**					0.367			
Right knee	22 (51.16%)	5 (35.71%)	9 (60.00%)	8 (57.14%)				
Left knee	21 (48.84%)	9 (64.29%)	6 (40.00%)	6 (42.86%)				
**Height**	156.22 ± 7.50	153.06 ± 6.65	156.90 ± 8.24	158.64 ± 6.84	0.154			
**Body weight**	68.07 ± 11.17	67.80 ± 8.75	68.50 ± 15.93	67.88 ± 7.25	0.963			
**BMI**	27.83 ± 3.63	28.97 ± 3.55	27.53 ± 4.32	27.01 ± 2.76	0.335			
**HKA angle**	9.02 ± 5.06	10.74 ± 5.61	9.03 ± 4.64	7.29 ± 4.67	0.188			
**Peak torque (Nm), Knee**								
Extension at 60 deg./sec	15.90 ± 9.48	12.67 ± 8.30	18.91 ± 10.81	15.91 ± 8.60	0.167			
Flexion at 60 deg./sec	16.54 ± 10.77	14.94 ± 8.04	20.83 ± 14.56	13.55 ± 6.96	0.452			
Extension at 120 deg./sec	13.10 ± 7.42	10.96 ± 6.56	16.59 ± 8.65	11.49 ± 5.71	0.060			
Flexion at 120 deg./sec	11.69 ± 6.23	10.41 ± 4.02	13.52 ± 8.61	10.99 ± 4.83	0.607			
**TUG test (sec.)**	12.99 ± 4.80	13.76 ± 5.53	13.31 ± 3.06	15.64 ± 3.73	0.208			
**SF-36 score**	46.56 ± 12.62	43.73 ± 11.33	48.71 ± 16.35	47.09 ± 9.15	0.599			
**WOMAC**	36.40 ± 14.37	36.86 ± 9.07	29.27 ± 11.14	43.57 ± 18.35	0.038*	0.170	1.000	0.047*

*BMI* Body mass index, *HKA* Hip-knee-ankle axis, *SF-36* Short-Form 36 Health Survey, *TUG* Timed up and go*, WOMAC* Western Ontario and McMaster Universities index

*p < 0.05

**Fig. 2:**
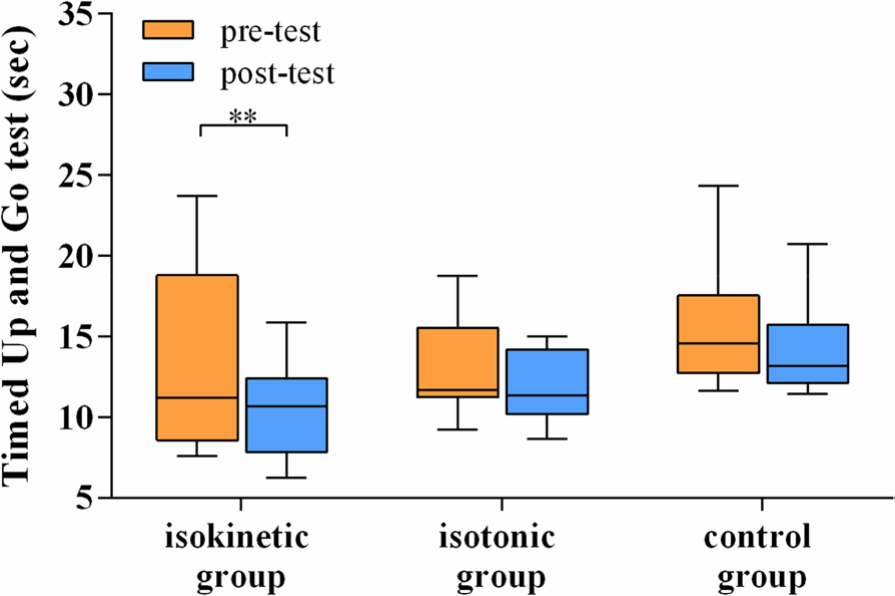
Box-plots of changes of Timed Up and Go test before and after different strengthening programs. The boundary of the lower whisker and upper whisker in the box-plot means the minimum and the maximum time taken in TUG, respectively

The data of the outcome measures before and after the 4 weeks of training are shown in Table 2. After 4 weeks of strengthening training after recruitment of patients, all of the peak torque measurements improved in both the isokinetic and the isotonic group, but only partial parameters of peak torque (knee extension and flexion at 60 deg./sec) did in the control group. However, as shown in Fig. 2, the time taken to perform the TUG test was significantly decreased in the isokinetic group only after 4-week training, but this phenomenon was not observed in the isotonic group. Furthermore, despite a significantly improved WOMAC index in all three groups, the total SF-36 score did not have a significant improvement in the control group. Only the physical domain of the SF-36 significantly improved in the control group patients.

**Table 2:** The outcome measures before and after four weeks of training or follow-up

	Isokinetic			Isotonic			Control		
	Pre-test	Post-test	*p* value	Pre-test	Post-test	*p* value	Pre-test	Post-test	*p* value
	Mean ± SD	Mean ± SD		Mean ± SD	Mean ± SD		Mean ± SD	Mean ± SD	
**HKA angle**	10.74 ± 5.61	3.53 ± 2.13	0.001**	9.03 ± 4.64	2.67 ± 1.97	0.001**	7.29 ± 4.67	3.02 ± 2.83	0.001**
**Peak torque (Nm), Knee**									
Extension at 60 deg./sec	12.67 ± 8.30	22.44 ± 12.65	0.006**	18.91 ± 10.81	33.67 ± 14.92	0.001**	15.91 ± 8.60	19.75 ± 8.00	0.035*
Flexion at 60 deg./sec	14.94 ± 8.04	22.01 ± 8.24	0.048*	20.83 ± 14.56	36.60 ± 15.55	0.001**	13.55 ± 6.96	15.06 ± 6.96	0.096
Extension at 120 deg./sec	10.96 ± 6.56	15.87 ± 7.40	0.016*	16.59 ± 8.65	26.74 ± 12.10	0.001**	11.49 ± 5.71	11.22 ± 4.01	0.754
Flexion at 120 deg./sec	10.41 ± 4.02	14.93 ± 5.47	0.016*	13.52 ± 8.61	23.61 ± 10.97	0.001**	10.99 ± 4.83	11.82 ± 3.52	0.346
**TUG test (sec.)**	13.76 ± 5.53	10.54 ± 2.90	0.003**	13.31 ± 3.06	12.39 ± 3.26	0.053	15.64 ± 3.73	14.76 ± 4.15	0.177
**SF-36 total score**	43.73 ± 11.33	57.24 ± 16.99	0.009**	48.71 ± 16.35	61.82 ± 15.21	0.012*	47.09 ± 9.15	49.05 ± 16.21	0.510
**Physical domain**	39.38 ± 14.85	52.80 ± 17.25	0.016*	43.17 ± 13.91	54.63 ± 16.55	0.012*	37.57 ± 10.42	43.30 ± 13.46	0.041*
Physical functioning	44.29 ± 25.33	57.14 ± 25.77	0.084	40.33 ± 20.57	61.00 ± 22.77	0.002**	41.43 ± 24.84	54.64 ± 19.85	0.032*
Physical role	5.36 ± 14.47	26.79 ± 35.98	0.026*	10.00 ± 22.76	23.33 ± 37.16	0.196	5.36 ± 10.65	5.36 ± 10.65	1.000
Bodily pain	55.00 ± 19.73	66.71 ± 14.15	0.041*	54.33 ± 19.51	73.83 ± 14.48	0.002**	46.43 ± 18.52	57.50 ± 16.35	0.125
General health	52.86 ± 18.68	60.57 ± 19.21	0.256	68.00 ± 19.53	66.33 ± 18.27	0.728	56.79 ± 15.52	55.71 ± 20.27	0.497
**Mental domain**	45.75 ± 13.55	61.31 ± 18.82	0.013*	54.26 ± 22.50	69.07 ± 17.87	0.026*	56.68 ± 14.99	54.50 ± 20.70	0.925
Vitality	48.93 ± 16.19	56.07 ± 20.86	0.092	59.67 ± 19.77	62.33 ± 20.08	0.430	57.86 ± 18.88	56.43 ± 17.70	0.443
Social functioning	61.68 ± 19.39	69.64 ± 19.44	0.134	58.33 ± 21.99	72.03 ± 16.90	0.020*	62.50 ± 25.00	56.25 ± 22.87	0.320
Emotional role	16.67 ± 36.40	59.52 ± 45.63	0.026*	37.78 ± 41.53	68.89 ± 42.66	0.111	45.24 ± 30.96	40.48 ± 45.63	0.796
Mental health	55.71 ± 10.92	60.00 ± 18.36	0.276	65.93 ± 21.78	72.32 ± 16.89	0.073	61.14 ± 18.72	64.86 ± 14.22	0.128
**WOMAC total score**	36.86 ± 9.07	21.36 ± 10.40	0.001**	29.27 ± 11.14	15.13 ± 11.15	0.001**	43.57 ± 18.35	33.79 ± 16.39	0.043*
Pain	7.64 ± 4.27	5.21 ± 3.12	0.025*	5.44 ± 2.79	3.56 ± 1.94	0.024*	9.00 ± 4.47	6.93 ± 2.34	0.046*
Stiffness	3.86 ± 0.95	2.43 ± 1.34	0.011*	2.89 ± 1.27	1.89 ± 1.05	0.071	3.64 ± 2.06	3.50 ± 1.34	0.623
Physical function	25.36 ± 6.37	13.71 ± 7.38	0.001**	16.56 ± 8.63	8.56 ± 6.77	0.008**	30.93 ± 12.41	23.36 ± 13.24	0.046*

*HKA* Hip-knee-ankle axis, *SF-36* Short-Form 36 Health Survey, *TUG* Timed up and go, *WOMAC* Western Ontario and McMaster Universities index

*p < 0.05, **p < 0.01

**Fig. 3:**
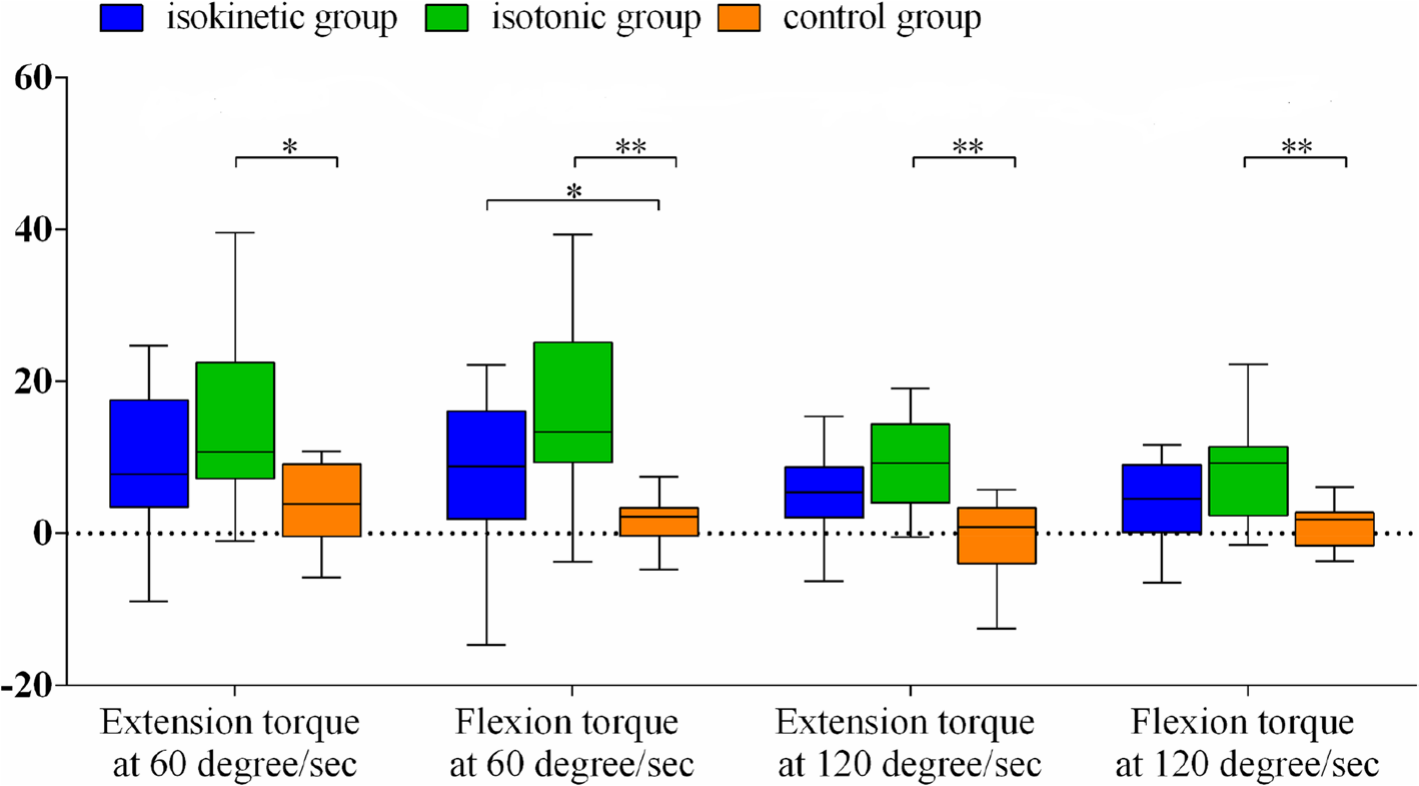
Box-plots of changes of peak torque of knee extension and flexion in various angular velocity after and before 4-week training programs in different groups; *p < 0.05, **p < 0.01

A comparison of the degree of improvement in the outcome measures is shown in Table 3. There were no significant differences in the time taken to perform the TUG test, SF-36 score, and WOMAC index among the three groups. Only the mental domain score differed among the three groups, but no significant difference could be established in the post hoc Bonferroni test between groups. Nevertheless, the peak torque of the knee significantly differed among the three groups. The post hoc Bonferroni test for the four parameters of peak torque showed a significant difference between the isotonic group and the control group, but not between isokinetic group and the control group. On average, the peak torque improved the most in the isotonic group before and after training, as shown in Fig. 3. Further comparisons of the amount of improvement on the outcome parameters between the IK and IT groups were shown in Table 4. However, there were no significant differences in all the parameters between the isokinetic and isotonic training in our study.

**Table 3:** The degree of improvement of outcome measures after 4-week training or follow-up

	Training mode			*p* value	Dunn-bonferroni post hoc		
	Isokinetic (IK) (*n* = 14)	Isotonic (IT) (*n* = 15)	Control (*n* = 14)		IK vs IT	IK vs control	IT vs control
**Peak torque (Nm), Knee**							
Extension at 60 deg./sec	9.77 ± 9.34	14.76 ± 11.29	3.84 ± 5.23	0.013*	0.862	0.209	0.011*
Flexion at 60 deg./sec	7.06 ± 11.11	15.77 ± 12.37	1.51 ± 3.51	0.001**	0.517	0.048*	< 0.001**
Extension at 120 deg./sec	4.91 ± 5.79	10.15 ± 9.39	−0.27 ± 4.85	0.001**	0.535	0.073	0.001**
Flexion at 120 deg./sec	4.51 ± 5.32	10.09 ± 8.77	0.83 ± 2.82	0.003**	0.456	0.146	0.002**
**TUG test (sec.)**	−3.22 ± 3.57	−0.93 ± 2.25	−0.88 ± 2.27	0.163			
**SF-36 total score**	13.51 ± 14.17	13.10 ± 16.10	1.95 ± 14.45	0.061			
**Physical domain**	13.43 ± 17.82	11.46 ± 15.17	5.73 ± 10.21	0.267			
Physical functioning	12.86 ± 31.61	20.67 ± 18.41	13.21 ± 20.53	0.622			
Physical role	21.43 ± 30.79	13.33 ± 36.43	0.00 ± 13.87	0.126			
Bodily pain	11.71 ± 18.63	19.50 ± 19.16	11.07 ± 26.63	0.582			
General health	7.71 ± 18.74	− 1.67 ± 21.10	− 1.07 ± 11.96	0.433			
**Mental domain**	15.56 ± 20.21	14.81 ± 23.18	− 2.18 ± 20.39	0.045*	1.000	0.109	0.078
Vitality	7.14 ± 14.77	2.67 ± 11.93	− 1.43 ± 18.23	0.310			
Social functioning	7.96 ± 17.39	13.70 ± 18.95	− 6.25 ± 32.43	0.051			
Emotional role	42.85 ± 54.59	31.11 ± 64.82	− 4.76 ± 45.02	0.054			
Mental health	4.29 ± 15.09	6.39 ± 11.77	3.71 ± 14.42	0.995			
**WOMAC total score**	−15.50 ± 10.18	− 14.13 ± 9.36	− 9.79 ± 15.23	0.501			
Pain	−2.43 ± 3.30	−1.89 ± 2.26	−2.07 ± 3.43	0.979			
Stiffness	−1.43 ± 1.60	−1.00 ± 1.41	− 0.14 ± 1.17	0.060			
Physical function	−11.64 ± 7.40	−8.00 ± 8.72	− 7.57 ± 12.35	0.327			

*SF-36* Short-Form 36 Health Survey, *TUG* Timed up and go, *WOMAC* Western Ontario and McMaster Universities index

*p < 0.05, **p < 0.01

**Table 4:** The comparisons of improvement after training between IK and IT group

	Isokinetic (IK) (*n* = 14)	Isotonic (IT) (*n* = 15)	Mean Difference (95% CI)	*p* value
**Peak torque (Nm), Knee**				
Extension at 60 deg./sec	9.77 ± 9.34	14.76 ± 11.29	−4.99 (− 12.92, 2.94)	0.285
Flexion at 60 deg./sec	7.06 ± 11.11	15.77 ± 12.37	− 8.70 (− 17.68, 0.28)	0.158
Extension at 120 deg./sec	4.91 ± 5.79	10.15 ± 9.39	− 5.24 (− 11.24, 0.76)	0.109
Flexion at 120 deg./sec	4.51 ± 5.32	10.09 ± 8.77	−5.58 (− 11.15, 0.00)	0.120
**TUG test (sec.)**	−3.22 ± 3.57	−0.93 ± 2.25	−2.29 (− 4.55, − 0.03)	0.102
**SF-36 total score**	13.51 ± 14.17	13.10 ± 16.10	0.40 (− 11.18, 11.99)	0.847
**Physical domain**	13.43 ± 17.82	11.46 ± 15.17	1.97 (− 10.61, 14.55)	0.554
Physical functioning	12.86 ± 31.61	20.67 ± 18.41	− 7.81 (− 27.35, 11.73)	0.689
Physical role	21.43 ± 30.79	13.33 ± 36.43	8.10 (− 17.7, 33.89)	0.506
Bodily pain	11.71 ± 18.63	19.50 ± 19.16	− 7.79 (− 22.20, 6.63)	0.366
General health	7.71 ± 18.74	−1.67 ± 21.10	9.38 (− 5.87, 24.63)	0.263
**Mental domain**	15.56 ± 20.21	14.81 ± 23.18	0.75 (− 15.88, 17.38)	0.949
Vitality	7.14 ± 14.77	2.67 ± 11.93	4.48 (− 5.72, 14.67)	0.272
Social functioning	7.96 ± 17.39	13.70 ± 18.95	−5.74 (− 19.63, 8.15)	0.474
Emotional role	42.85 ± 54.59	31.11 ± 64.82	11.74 (− 34.09, 57.57)	0.852
Mental health	4.29 ± 15.09	6.39 ± 11.77	−2.10 (− 12.37, 8.17)	0.991
**WOMAC total score**	−15.50 ± 10.18	−14.13 ± 9.36	−1.37 (− 8.81, 6.08)	0.613
Pain	−2.43 ± 3.30	−1.89 ± 2.26	− 0.54 (− 3.16, 2.08)	0.912
Stiffness	−1.43 ± 1.60	−1.00 ± 1.41	− 0.43 (− 1.79, 0.93)	0.453
Physical function	−11.64 ± 7.40	−8.00 ± 8.72	−3.64 (− 10.69, 3.40)	0.164

Mann-Whitney U test. *p < 0.05, **p < 0.01.; 95% CI: 95% Confidence Intervals

## Discussion

Our study findings showed that isokinetic training during the early stage of TKR resulted in the most significant improvement in objective functional performance of the lower limbs, and the time taken to perform the TUG test, although isotonic training achieved an even better increase in strength. Moreover, home isometric education significantly improved self-perceived physical function and pain in spite of a lack of significant strength improvement.

The primary outcome measure of this study, the time taken to perform the TUG, was significantly lowered after training while comparing to the TUG before training in the isokinetic training group only (*p* = 0.003), but not in other two groups. (Table 2 and Fig. 2). Although the degree of improvement did not significantly differ among the three groups, only that of the isokinetic group exceeded 2.27 seconds, the minimal detectable change (MDC) for patients receiving TKR according to previous literature [18]. The results of our study are compatible with a similar study [8], which compared the training effect among untrained men who received isokinetic, isotonic, and isometric strengthening exercise. The aforementioned study also showed that only the isokinetic group achieved a significant improvement in the triple-hop-distance test. Isokinetic training on shoulder exercise has been proven to be a more effective method of stimulating muscle activation [19], and previous research has shown that it is associated with less training-induced muscular injury and functional limitation [20]. However, no significant improvement in TUG was observed in the isotonic training group in our study, which differs from the finding of a study by Petterson, et al. [21]. A possible explanation for this discrepancy may be that their program lasted for 6 weeks, and their outcomes were assessed 3 months after TKR. In contrast, our programs were conducted for only 4 weeks, and the outcomes were assessed only 2 months after TKR. A longer period of isotonic training could have resulted in an improvement in TUG performance in our study. However, only four-week isokinetic training program gain the significant improvement of the time taken for TUG test more than the MDC after early stage of TKR in this research.

Our study revealed more strength gains in the isotonic group than in the other two groups, and this result is compatible with a review article [22], which concluded that the isotonic mode of strengthening leads to greater strength gains than the isokinetic mode. In our study, both the isokinetic and the isotonic groups showed significant improvement in peak torque strength, though the degree of improvement was not significantly different. The result of our study is consistent with findings reported by Remaud, et al. [23], which also showed significant strength improvement in both groups without a significant difference in the degree of improvement. While the control group in their study similarly showed no significant improvement as our control group, which only received home isometric exercise instructions, also showed no significant improvement in strength. Isometric exercise is considered an efficient method of building muscle strength. However, according to a review article [24], the training intensity should be at 70–75% of maximum voluntary contraction (MVC) to elicit muscular hypertrophy, and 80–100% MVC to increase maximal strength. Participants in our control group were instructed to perform isometric exercise by maintaining the knee extension posture while seated. Although this isometric exercise is the most well-known home exercise to improve the strength of the quadriceps muscle, as advocated by healthcare givers, the training intensity is apparently far from sufficient to induce strength gains, which may explain the lack of significant improvement in knee strength in the control group of our study. Another reason may be related to the compliance of this group of patients. Without personal supervision, as provided when performing hospital-based isokinetic or isotonic exercise, the compliance of patients doing exercise training at home may be much lower than that of the other two groups.

While there was no apparent strength gain in the home exercise control group, they still had significantly improved physical functioning score in the SF-36 and pain amelioration in the WOMAC. A meta-analysis of studies with high methodological quality also showed that home-based exercise could significantly improve pain and function in individuals with knee OA [25]. Furthermore, patients in both the isokinetic and the isotonic groups showed more improved items in the SF-36 than the control groups, with significantly improved physical domain scores, mental domain scores, and total SF-36 scores. However, another study revealed the minimal clinically important differences (MCIDs) for WOMAC and SF-36 after TKR were 15 and 10, respectively [26]. In our study, both isokinetic and isotonic groups had improved SF-36 scores beyond the MCID, but only the isokinetic group had an improved WOMAC score above the MCID. In two meta-analyses that compared the results between hospital-based and home-based rehabilitation programs, both showed no significant differences in mobility, pain, and function between the two groups [27, 28]. One meta-analysis revealed a better pain score in the hospital-based group at 52 weeks [27]. The results of our study are compatible with the two meta-analyses.

The main strength of this study was the single-blinded and randomized controlled design that allowed a head-to-head comparison of results among patients who underwent TKR and then received one of three distinct postoperative rehabilitation programs. Furthermore, the participants received the TKR surgery performed by a single surgeon, which avoided the confounding factor of diverse surgical techniques used by different surgeons. However, there were at least three limitations in this study. First, the level of daily activity and exercise habits were not recorded in our participants. Any change in the activity of daily life could have confounded the outcome measures of our study. In order to minimize this confounding effect, we reminded our participants to refrain from abrupt changes in daily activities or exercise habits during the 4 weeks of the study when they signed the informed consent form. Second, as mentioned above, the intensity of the home isometric exercise could be far from sufficient to elicit strength gains. Therefore, patients in this group could only be regarded as the control group in this study, as they received only routine home exercise education, just as other typical TKR patients receive elsewhere. Third, there seems to be more females in the isokinetic group comparing to those in the other two groups, which may have some influences on theirs functional recovery due to this gender differences. However, the gender proportion in the three groups was not significantly different after statistical examination (*p* = 0.204). Finally, this was only a single-center study, which may limit the generalizability of the findings to other patient groups. Further larger scale, multi-centered studies should be designed in the future to better validate the results of this study.

## Conclusion

Our study confirmed the benefits of isokinetic exercise training in old participants receiving TKR with respect to strength gains, functional performance of lower limbs in terms of TUG, and quality of life. Although isotonic exercise could elicit more strength gains, only isokinetic training was capable of enhancing the performance of TUG. Therefore, both the isokinetic and isotonic exercise are recommended for patients receiving TKR to further facilitate either recovery of lower limb functions or strength gains.

## Data Availability

The datasets used and/or analyzed during the current study available from the corresponding author on reasonable request.

## Abbreviations

TKR: Total knee replacement
OA: Osteoarthritis
IK: isokinetic
IT: isotonic
TUG: Timed Up and Go test.
SF-36: Short-Form 36 Health Survey
WOMAC: Western Ontario and McMaster Universities Arthritis index
ANOVA: analysis of variance
MVC: maximum voluntary contraction
MCIDs: minimal clinically important differences
